# Understanding calcium functionality by examining growth characteristics and structural aspects in calcium-deficient grapevine

**DOI:** 10.1038/s41598-022-06867-4

**Published:** 2022-02-25

**Authors:** Shuyan Duan, Chengjun Zhang, Shiren Song, Chao Ma, Caixi Zhang, Wenping Xu, Bhaskar Bondada, Lei Wang, Shiping Wang

**Affiliations:** 1 School of Agriculture and Biology, Shanghai Jiao Tong University, Shanghai, 200240 People’s Republic of China; 2 Department of Horticulture, Washington State University Tri-Cities, Richland, WA 99354 USA; 3 Institute of Agro-Food Science and Technology/Key Laboratory of Agro-Products Processing Technology of Shandong, Shandong Academy of Agricultural Sciences, Jinan, 250100 People’s Republic of China

**Keywords:** Cell biology, Ecology, Plant sciences

## Abstract

This study characterized growth characteristics and cellular details employing microscopy techniques in hydroponically-grown Ca^2+^-sufficient and Ca^2+^-deficient grapevines (*Vitis vinifera*) in a glasshouse. The Ca^2+^-deficient vines exhibited significant reductions in shoot length, shoot and trunk fresh weights, leaf area, chlorophyll, which eventually led to drooping, yellowing, and chlorosis of leaves. Roots were less dense and primarily dark and necrotic. Furthermore, their xylem vessels were small, polygonal, and appeared to be collapsed yet increased in number and developed lateral roots. Despite such alterations, the anatomical organization of leaves was not affected, yet they developed with more xylem vessels with thick walls and lignin in their mesophyll and vascular tissues. The chloroplasts in internodes’ chlorenchyma, phloem, and cambium underwent significant ultrastructural modifications. The concentrations of macro and micronutrients varied significantly among the roots, trunk, canes, and leaves, including the growth characteristics. These structural and growth modifications of calcium deficiency enable us to understand better the link between the symptoms and functions and for a holistic understanding of Ca^2+^ functionalities.

## Introduction

Next to water, the availability and plants’ ability to acquire soil mineral nutrients determine our sustainable development and global health^1,2^. Arguably, in this context, none other than calcium (Ca^2+^), the third most abundant metal in nature, has been influential and distinguishable in many ways vis-à-vis the rest of the nutrients for many reasons^3^. For instance, it regulates almost all our bodily and plant functions by serving both as a nutrient and a messenger^4^. Despite its prominence and the tsunamic corpus of information accumulated over the years, its most explicit contribution to plant metabolism is yet to emerge^5^. The complex process of its uptake entailing transport pathways, their diverse absorption abilities, and the lack of its redistribution accounts for such ambiguity and it being an intractable element^2,5–7^. As a result, Ca^2+^ nutritional studies never yielded reliable results, leaving many obscurities in its role as an essential element^2,3,5,6^. Nevertheless, since it directed life to evolve and conserve by promoting and regulating metabolic functions, a strong socio-economical driving force to understand its precise function persists^4^.

To gain a realistic understanding of how Ca^2+^ influences the growth and structure of plants, including their productivity^2,7^, we need to reveal how Ca^2+^ transport and function are integrated from the whole plant to the subcellular level in different species^8^. Progress in this endeavor requires in-depth characterization and fundamental understanding of the symptomatology of organs in different plants at the whole plant and cellular levels by conducting starvation studies under controlled conditions^1,9,10^. Unlike other nutrients^11,12^, the links between Ca^2+^ deficiency and its symptomatology in different organs, including the complex interaction among other nutrients in its absence in various crops, are paradoxical, hence the limited understanding of its functionality^5^. This conundrum ensues because tissue requirements for Ca^2+^ are genetically controlled, expressing at different extents^7,13^. Consequently, its levels vary with stages of growth, among and within plant species^5^, between monocots and dicots^13^, and among genotypes of the same species^8^. Such wide variations result in uneven distribution among and within the plant parts^7,14^, and heterogeneous storage across cell types^15^, causing Ca^2+^ deficiency symptoms to differ from organ to organ, species to species, and among different genotypes of the same species^16^.

Given the challenge of deriving functional insights from the model plant, *Arabidopsis*^17^, the logical course of action to account for such wide variations is to document Ca^2+^ deficiency-related responses by sifting through symptoms of various tissues at cellular levels in each species and genotype^1^. Symptoms, the purest, truest, and the most tangible form of physiological response in advance to a harmful stimulus caused by abiotic and biotic factors mirror the abnormal function reflecting its role in the structural décor and metabolism. Based on this portrayal, the symptomatology conceptualizes functional roles using the imagery of nutrient deficiency symptoms. Typically, mineral nutrient deficiency is predicted and rectified through foliar analysis followed by fertilization^1^. Concerning Ca^2+^, such remedial measures present a daunting task because of its unique properties^5,18^. Consequently, to better understand the physiological functionalities of Ca^2+^, it is necessary to utilize deficiency symptoms and structural modifications^1^. These visual imageries imbued with the ability to reveal physiological insights provide us with a global perspective of plant function specific to the concerned nutrient^1,19,20^. Thus, the most articulate approach to determine the decisive physiological roles of Ca^2+^ is to integrate the directness of symptomatology with growth characteristics. That is because linking nutrient deficiency symptoms with physiological functions offers the simplest yet wide-reaching means capable of explaining and predicting the Ca^2+^ status in crop plants with high genetic diversity^1^. Because of this reason, the Ca^2+^ deficiency symptoms, relatively appearing first and very early^9,21,22^, have been investigated^4,9,19,20,23^ and used as diagnostic criteria for plants and soils^1,24^. This knowledge is acutely needed, especially now to sustain plant productivity against climate-change-induced challenging conditions such as mineral stress, water scarceness, increased groundwater salinity, soil pests build-up, etc.^5^. Our recent paper^10^ has covered some of this information by examining the physiology and carbohydrate metabolism (enzymes involved in photosynthesis and sugar metabolism) in Ca^2+^-sufficient and Ca^2+^-deficient grapevines. This study aims to comprehend the Ca^2+^ deficiency symptomatology related to growth, nutrient distribution, and morpho-anatomical and ultrastructural alterations of root and shoot systems. We chose grapevine because of its global socio-economic impact^25^, grown under Ca^2+^-deficient soil conditions^13^.

## Results

### Effects of Ca^2+^-deficiency and Ca^2+^-sufficiency on growth characteristics and leaf anatomy

**Figure 1:**
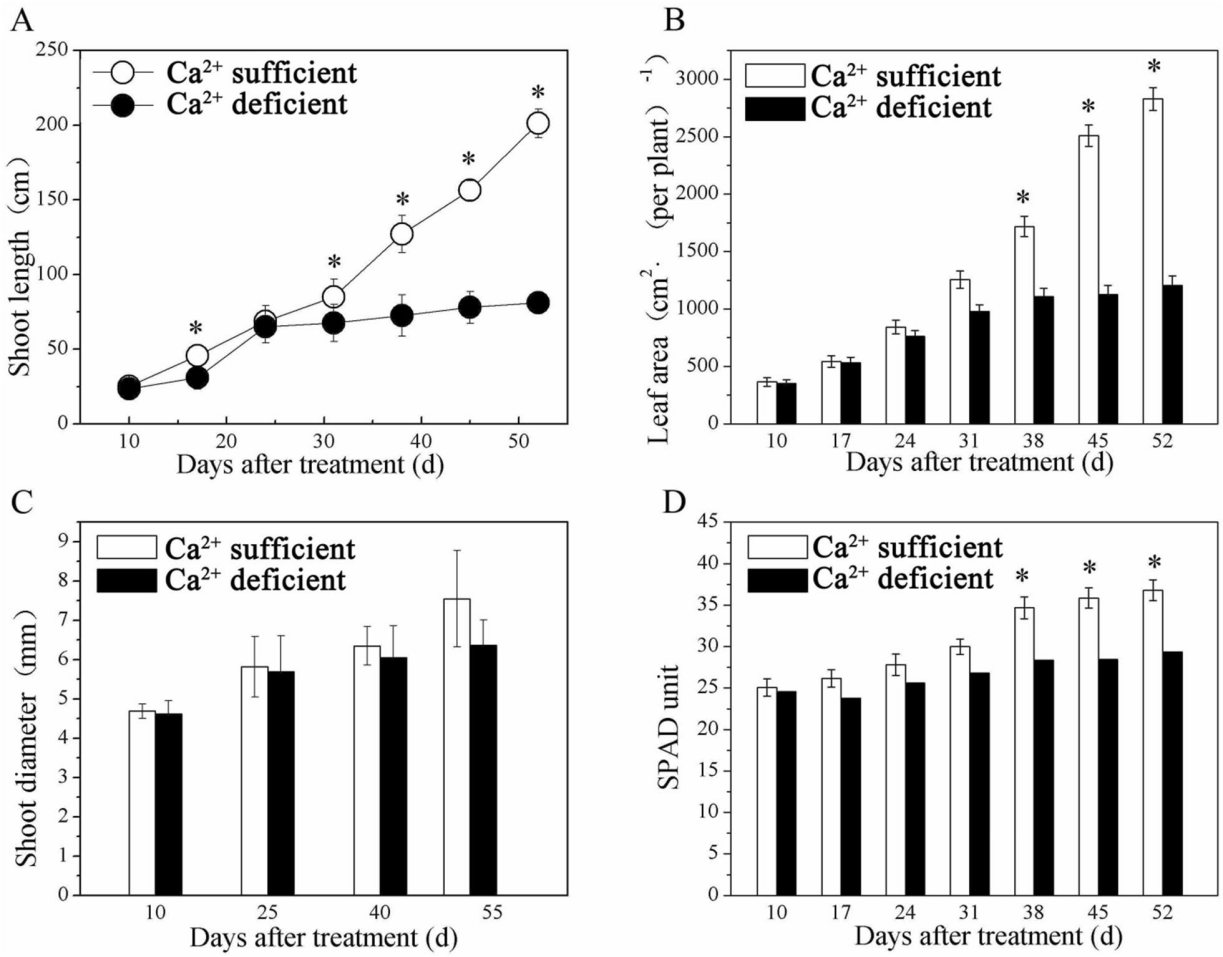
Shoot length (**A**), leaf area (**B**), shoot (stem) diameter(**C**), and (**D**) chlorophyll of Ca^2+^ deficient and Ca^2+^ sufficient vines. Within each graph, bars (mean ± SE) sharing a common letter are not significantly different according to Student’s *t* test at P < 0.05. Values are means of 3 replications.

**Figure 2:**
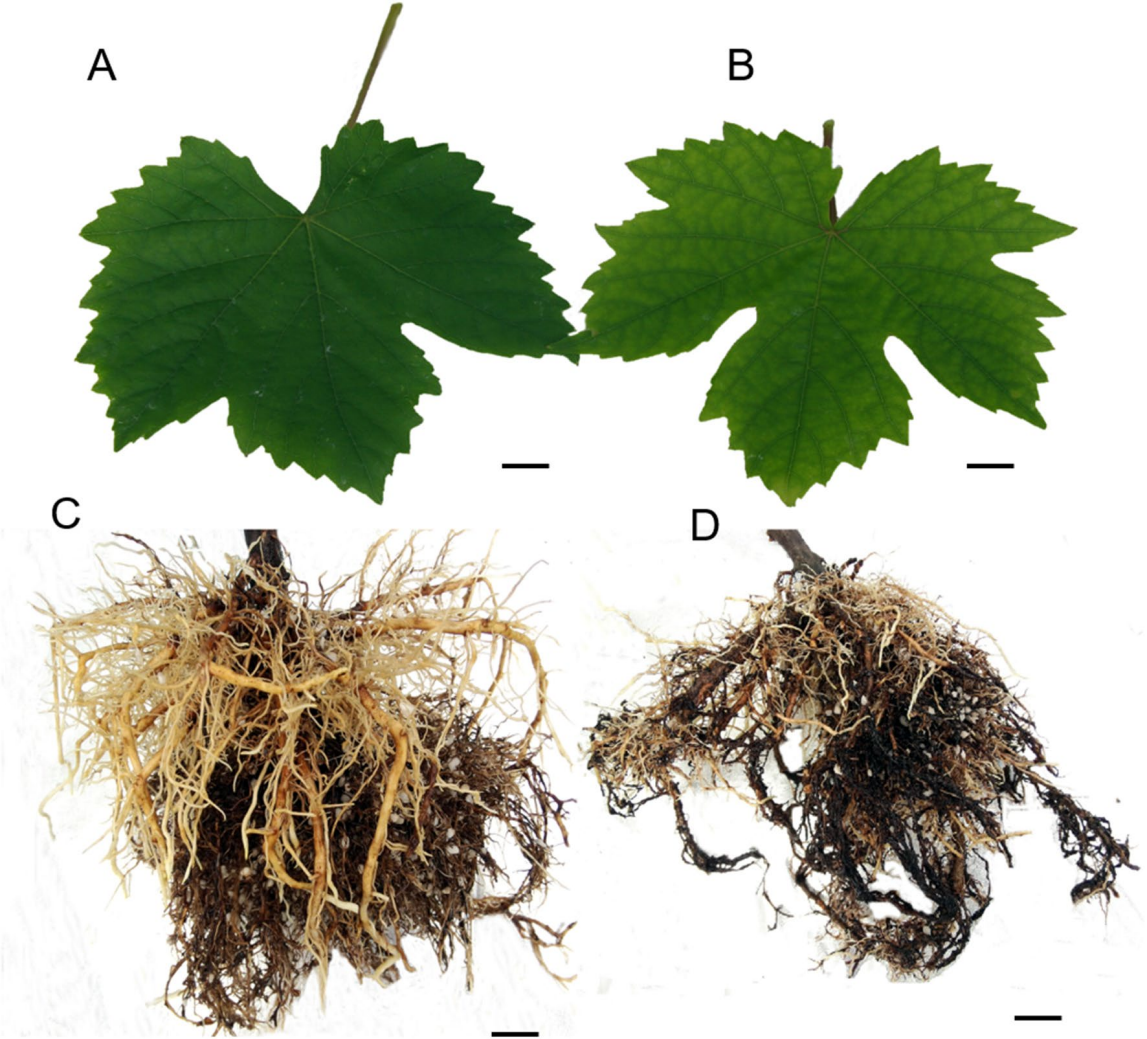
Effects of calcium supply on the morphology of leaf and roots at 24 DAT (days after treatment). (**A**) Healthy leaves with sufficient Ca^2+^, (**B**) Ca^2+^-deficient leaves expressed symptoms at 24 DAT and started to senesce at 52 DAT, (**C**) Healthy roots with sufficient Ca^2+^, and (**D**) Ca^2+^-deficient roots showing necrosis and reduced density. Scale bars: 1 cm (**A**–**D**).

**Figure 3:**
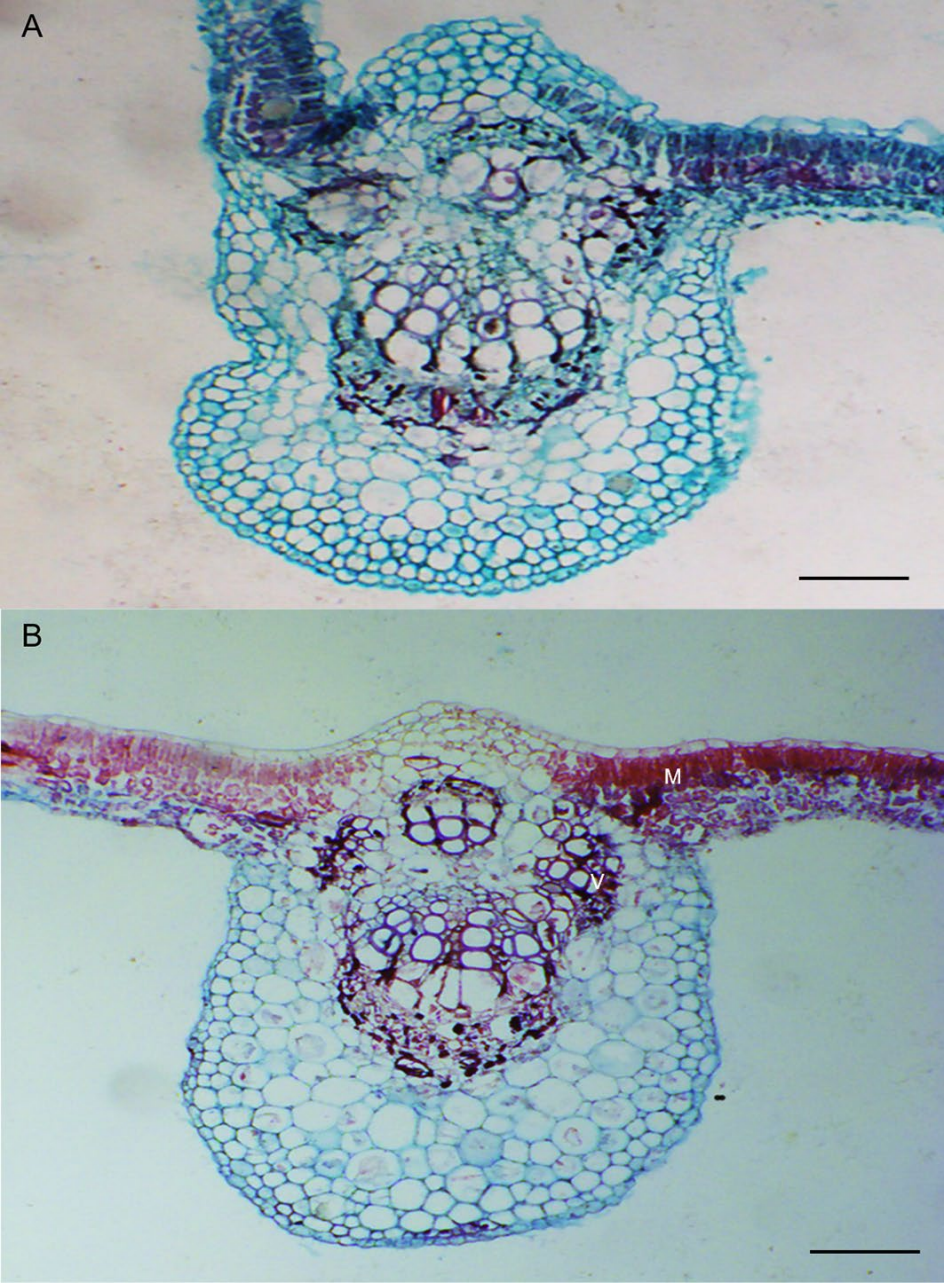
Transverse light micrographs of (**A**) Ca^2+^ sufficient leaves, (**B**) Ca^2+^ deficient leaves showing typical organization of tissues in the lamina and the vein. Notice that the cells are lignified in the Ca^2+^ deficient leaves as indicated by the intense staining of the vasculature and mesophyll tissues. Scale bars: 50 µm (**A**), 100 µm (**B**). *M* mesophyll, *V* vasculature.

Shoot symptoms included significant reductions in shoot length (Fig. 1A), leaf area (Fig. 1B), and chlorophyll level (Fig. 1D) in the Ca^2+^-deficient vines with no difference in the stem diameter between Ca^2+^-sufficient and Ca^2+^-deficient vines (Fig. 1C). The leaves of Ca^2+^-sufficient vines were healthy and chlorophyllous (Fig. 2A). The leaves of Ca^2+^-deficient vines were droopy, yellowish, and chlorotic (Fig. 2B). These features corresponded to less dense and primarily dark and necrotic roots (Fig. 2D) as opposed to the healthy Ca^2+^-sufficient roots (Fig. 2C). Furthermore, except for the roots and the canes, the dry and fresh weights of all organs declined in the Ca^2+^-deficient vines (Table 1). Akin to stem diameter, leaf anatomical organization did not differ between Ca^2+^-deficient and Ca^2+^-sufficient vines (Fig. 3A,B). Also, leaves from both treatments exhibited similar morpho-anatomy having asymmetries in their proximo-distal, adaxial–abaxial (dorsal–ventral), and mediolateral axes to become dorsiventral laminar structures in which the photosynthetic, supportive, stomatal, and leaf vasculature cell types differentiated in specific positions (Fig. 3A,B). Nonetheless, Ca^2+^-deficient leaves developed with more lignin and xylem vessels with thick cell walls than the Ca^2+^-sufficient leaves (Fig. 3B; Table 2). These reductions in shoot length, shoot and trunk fresh weights, leaf area, and chlorophyll coupled with necrotic roots eventually led to drooping, yellowing, and chlorosis of leaves in the Ca^2+^-deficient vines.

**Table 1:** The fresh (FW) and dry weights (DW) of grapevine’s organs at 52 DAT.

Organs	FW (g)		DW (g)	
	Ca^2+^-sufficiency	Ca^2+^-deficiency	Ca^2+^-sufficiency	Ca^2+^-deficiency
Root	30.88 ± 2.05	34.20 ± 1.51*	4.75 ± 0.52	5.58 ± 0.43*
Trunk	32.38 ± 4.12*	21.96 ± 1.53	6.44 ± 0.36*	4.55 ± 0.51
Cane	12.93 ± 0.86	18.08 ± 0.98*	4.63 ± 0.34	6.80 ± 0.58*
Stem	10.45 ± 0.52*	6.30 ± 0.29	1.54 ± 0.16	0.79 ± 0.05
Leaf	37.31 ± 3.21*	22.72 ± 3.18	8.36 ± 0.65*	5.50 ± 0.45
Petiole	10.18 ± 0.81*	5.95 ± 0.64	2.80 ± 0.08*	0.86 ± 0.09
Total	134.12 ± 10.92*	109.20 ± 8.39	28.51 ± 1.34	28.13 ± 3.64

*Means within a row followed by the same letter are not significantly different according to Students’ *t* test (P < 0.05).

**Table 2:** Leaf vessel morphology of Ca^2+^-sufficient and Ca^2+^-deficient grapevines.

Treatment	Tissues	Diameter	Area	Number	Wall thickness
Ca sufficiency	Major vein	29.28 ± 6.35*	631.07 ± 29.75*	31 ± 3.61	2.26 ± 0.29
Ca deficiency		20.17 ± 3.39	403.20 ± 25.80	47 ± 7*	5.27 ± 0.63*
Ca sufficiency	Lateral vein	11.06 ± 2.95	77.1 ± 34.93	8 ± 0.58	1.95 ± 0.37
Ca deficiency		9.66 ± 2.02	141.66 ± 22.42*	12 ± 0.58*	2.96 ± 0.50*

*Means within a column followed by the same letter are not significantly different according to Students’ *t* test (P < 0.05).

### Ultrastructural changes in the cambium, phloem, and chloroplasts of Ca^2+^-deficient and Ca^2+^-sufficient grapevine stem

**Figure 4:**
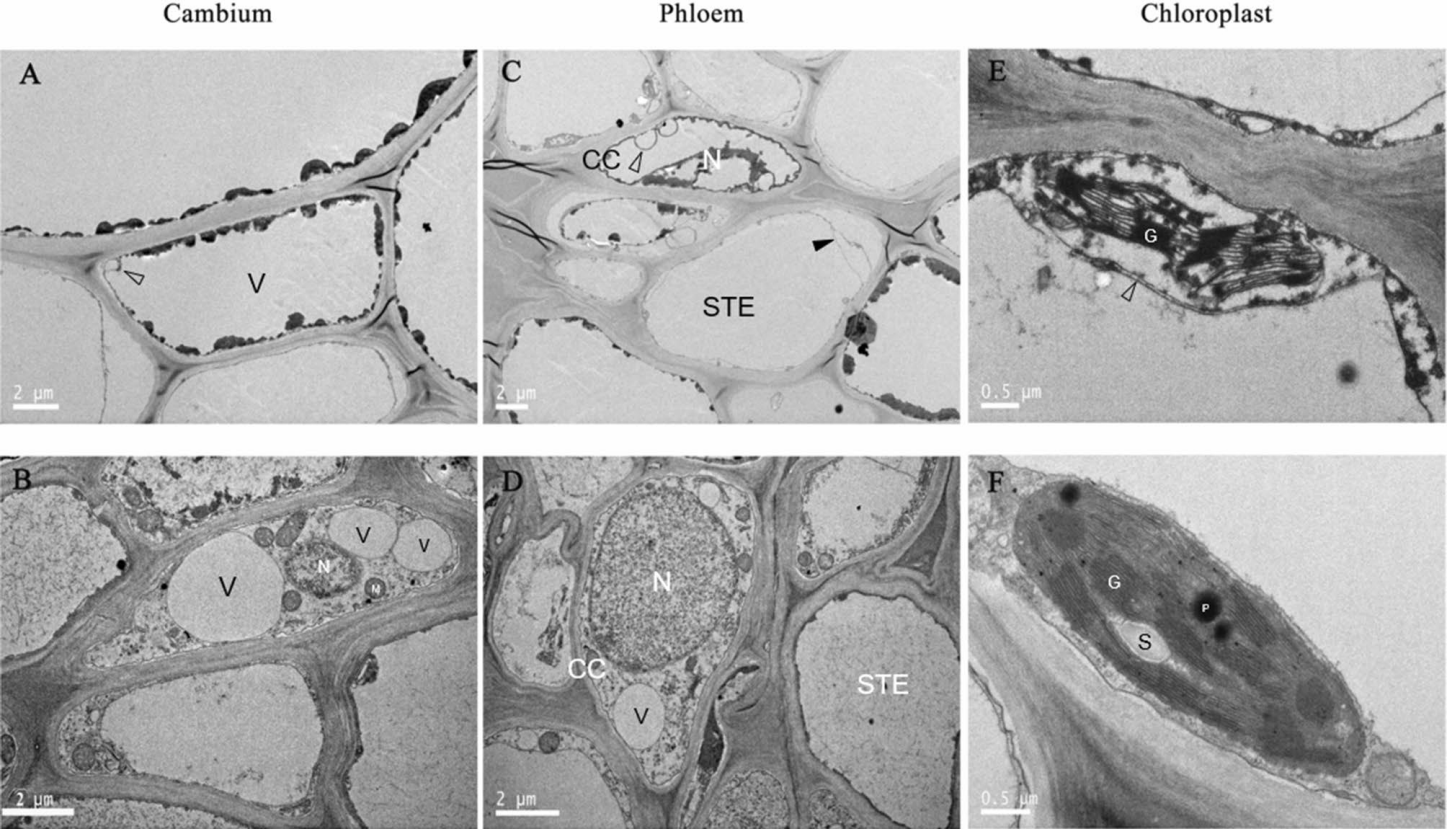
Transmission electron micrographs of (**A**) cambium, (**C**) phloem, and (**E**) chloroplast at 24 DAT of Ca^2+^ deficient stems; (**B**) cambium, (**D**) phloem, and (**F**) chloroplast at 24 DAT of Ca^2+^ sufficient stems. Scale bars: 2 μm (**A**–**D**), 0.5 μm (**E**,**F**). *CC* companion cells, *G* grana, *N* nucleus, *P* plastoglobuli, *S* starch, *SRE* sieve tube element, *V* vacuole. The arrowhead indicate membrane invagination (protuberance) in (**A**) and (**C**), and the closed arrowhead in (**C**) indicates membrane separation from the cell wall.

The stem diameter was not affected despite increased xylem vessels (Table 3). Nonetheless, the ultrastructure of tissues in its internodes, such as the chlorenchyma, phloem, and cambium, underwent structural modifications (Fig. 4). The Ca^2+^-deficient cambium cells were characterized by a large central vacuole surrounded by a parietal layer of dense cytoplasm that was electron opaque with protuberances (Fig. 4A). On the other hand, the characteristics observed in the Ca^2+^-sufficient cambium revealed that the organelles are densely and uniformly distributed throughout the cytoplasm (Fig. 4B). Instead of exhibiting one large vacuole, three large vacuoles coupled with mitochondria, and an almost spherical nucleus, more or less median position within the cell, were the main ultrastructural features in the Ca^2+^ sufficient cambium (Fig. 4B). Sieve elements and companion cells comprised the phloem. Companion cells in the Ca^2+^-deficient phloem were characterized by large and small vacuoles surrounded by a parietal layer of dense cytoplasm that was electron opaque with protuberances (Fig. 4C). The electron-dense material in the center appeared to be remnants of a degenerated nucleus (Fig. 4C). In the Ca^2+^-sufficient phloem, the companion cells were conspicuous by their electron-dense appearance and developed with typical cellular components such as a nucleus, small vacuoles, and mitochondria (Fig. 4D). The plasmalemma in the Ca^2+^-deficient sieve tube elements is folded at places and partially separated from the wall (Fig. 4C), unlike the Ca^2+^-sufficient sieve tube element wherein it was intact lining the cell wall (Fig. 4D). Furthermore, the lumen of the Ca^2+^-deficient sieve tube element was translucent (Fig. 4C) as opposed to electron-dense with granular material in the Ca^2+^-sufficient sieve tube element (Fig. 4D).

**Table 3:** Stem vessel morphology of Ca^2+^-sufficient and Ca^2+^-deficient grapevines.

Treatment	Diameter	Area	Number	Cell wall thickness
Ca sufficiency	8.36 ± 1.84*	77.8 ± 10.21*	12 ± 2.64	0.98 ± 0.15
Ca deficiency	5.91 ± 0.97	41.27 ± 8.79	26.33 ± 3.11*	0.87 ± 0.12

*Means within a column followed by the same letter are not significantly different according to Students’ *t* test (P < 0.05).

The chloroplast shape was somewhat distorted, yet the chloroplast envelope (inner and outer membranes) remained intact in the Ca^2+^-deficient stem’s chlorenchyma (Fig. 4E). Despite this membrane wholeness, the grana and stroma lamellae were few and were poorly developed, with the grana being pulled away from the envelope and mostly confined to the center of the chloroplast, and showed no plastoglobuli (Fig. 4E). Another important feature was the clustering of stroma lamellae (Fig. 4E). These features contrasted with the chloroplasts of Ca^2+^-sufficient stem’s chlorenchyma (Fig. 4F). They showed the typical lens-shaped chloroplast and ultrastructure with fully developed grana, stroma lamellae, plastoglobuli, and starch granules (Fig. 4F). It is evident from the examination above that Ca^2+^ deficiency causes significant ultrastructural changes in plant organelles and vascular tissues, including cambium, as observed in this study.

### Changes in the nutrient concentrations in roots, trunks, canes, and leaves of Ca^2+^-deficient and Ca^2+^-sufficient grapevines

**Figure 5:**
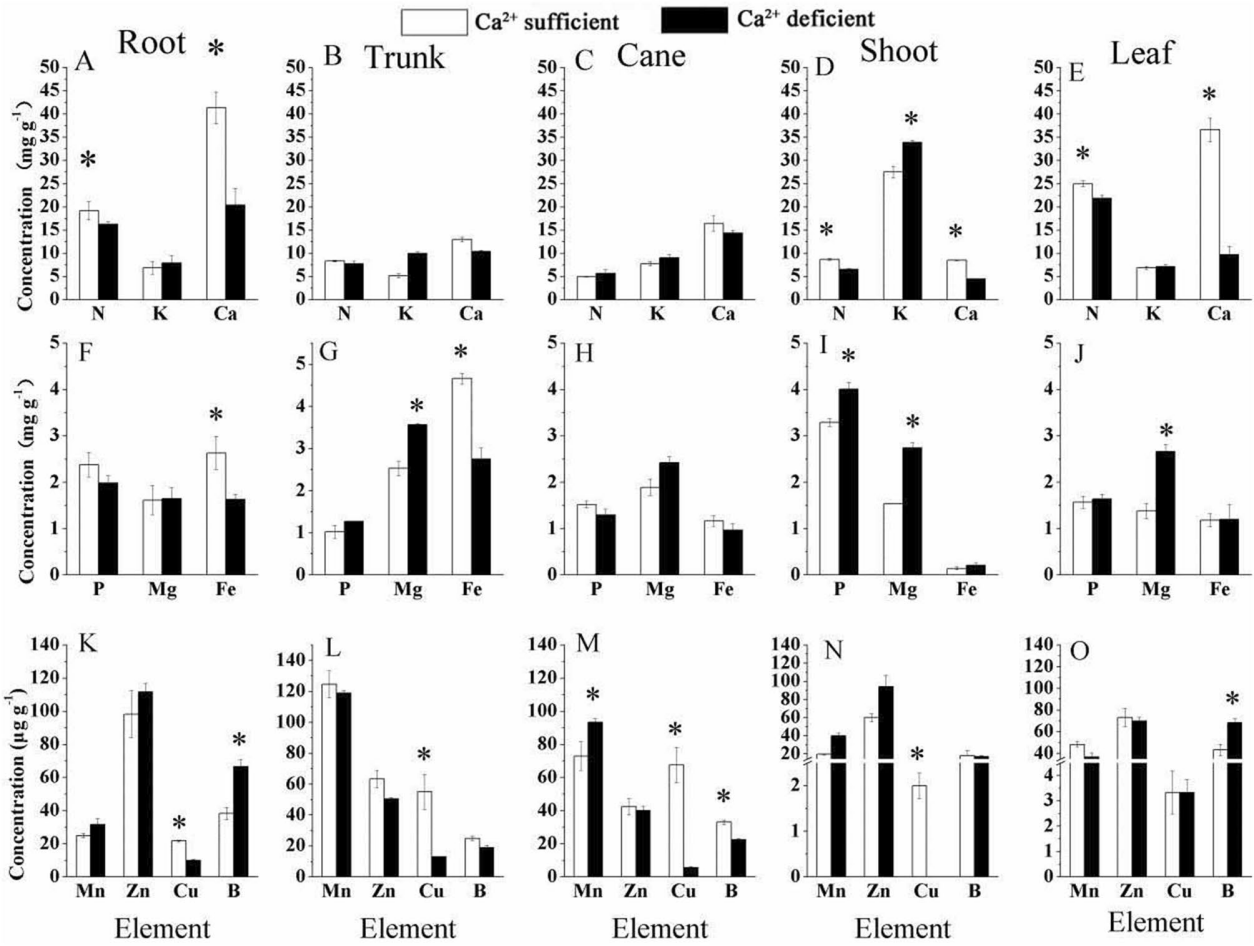
The macro and micro-nutrients concentration of root, trunk, cane, shoot (stem), and leaf in Ca^2+^ deficient and Ca^2+^ sufficient vines (**A**–**O**). Roots (**A**,**F**,**K**); trunk (**B**,**G**,**L**), cane (**C**,**H**,**M**); stem (**D**,**I**,**N**); and leaf (**E**,**J**,**O**). Within each graph, bars (mean ± SE) sharing a common letter are not significantly different according to Student’s *t* test at P < 0.05.

The nutrient concentrations, N, P, K, Ca, Mg, Fe, Mn, Zn, Cu, and B, varied among the roots, trunk, canes, and leaves (Fig. 5). For instance, Ca^2+^-deficiency-induced significant increases in the levels of B in roots (Fig. 5K), Mg in the trunk (Fig. 5G), Mn in canes (lignified stem with secondary growth) (Fig. 5M), K (Fig. 5D), P (Fig. 5I), and Mg (Fig. 5I) in the green stem, and Mg (Fig. 5J) and B (Fig. 5O) in leaves. In the Ca^2+^-sufficient vines, significant increases were found for Fe (Fig. 5G) and Cu (Fig. 5L) in the trunk, Cu (Fig. 5M) and B (Fig. 5M) in the canes, N (Fig. 5D), Ca (Fig. 5D), and Cu (Fig. 5N) in green stems, and N (Fig. 5E) and Ca (Fig. 5E) in the leaves. From the above uptake patterns, it is clear that the concentrations of macro and micronutrients vary significantly among different organs of the Ca^2+^ deficient vines.

### Root anatomical changes in Ca^2+^-deficient and Ca^2+^-sufficient grapevines

**Figure 6:**
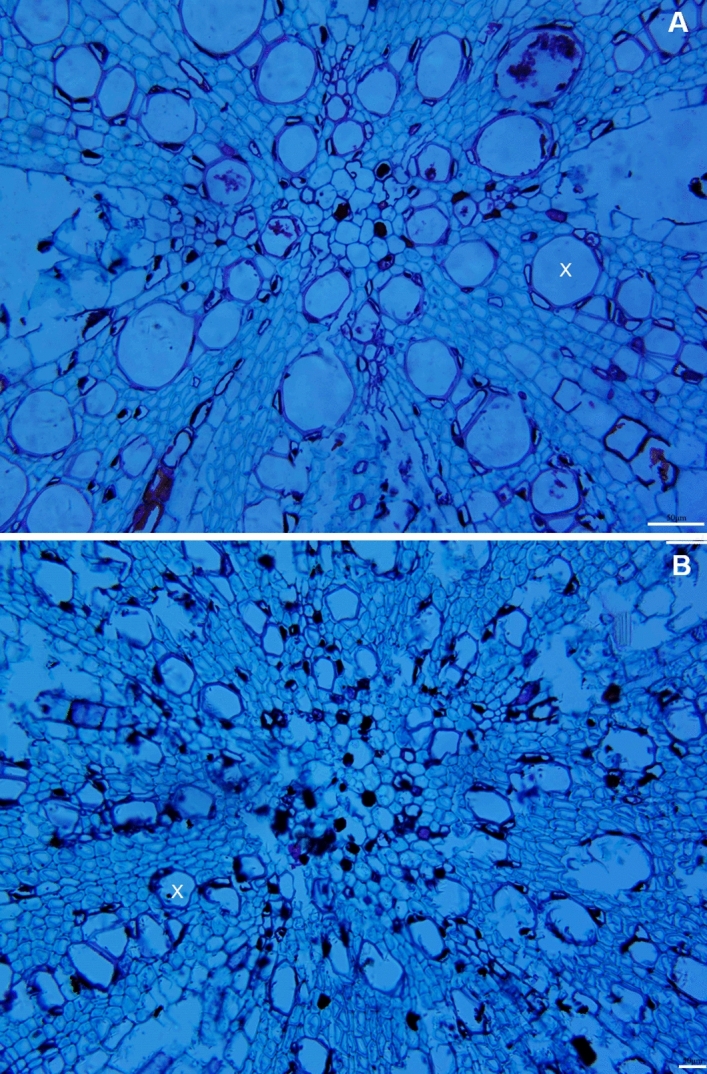
Transverse light micrographs of (**A**) Ca^2+^-sufficient and (**B**) Ca^2+^-deficient roots showing xylem vessels. As opposed to large and circular xylem vessels, they were small, polygonal, and appeared to be collapsed in the Ca^2+^-deficient roots. Scale bars: 50 µm (**A**,**B**). *X* xylem.

**Figure 7:**
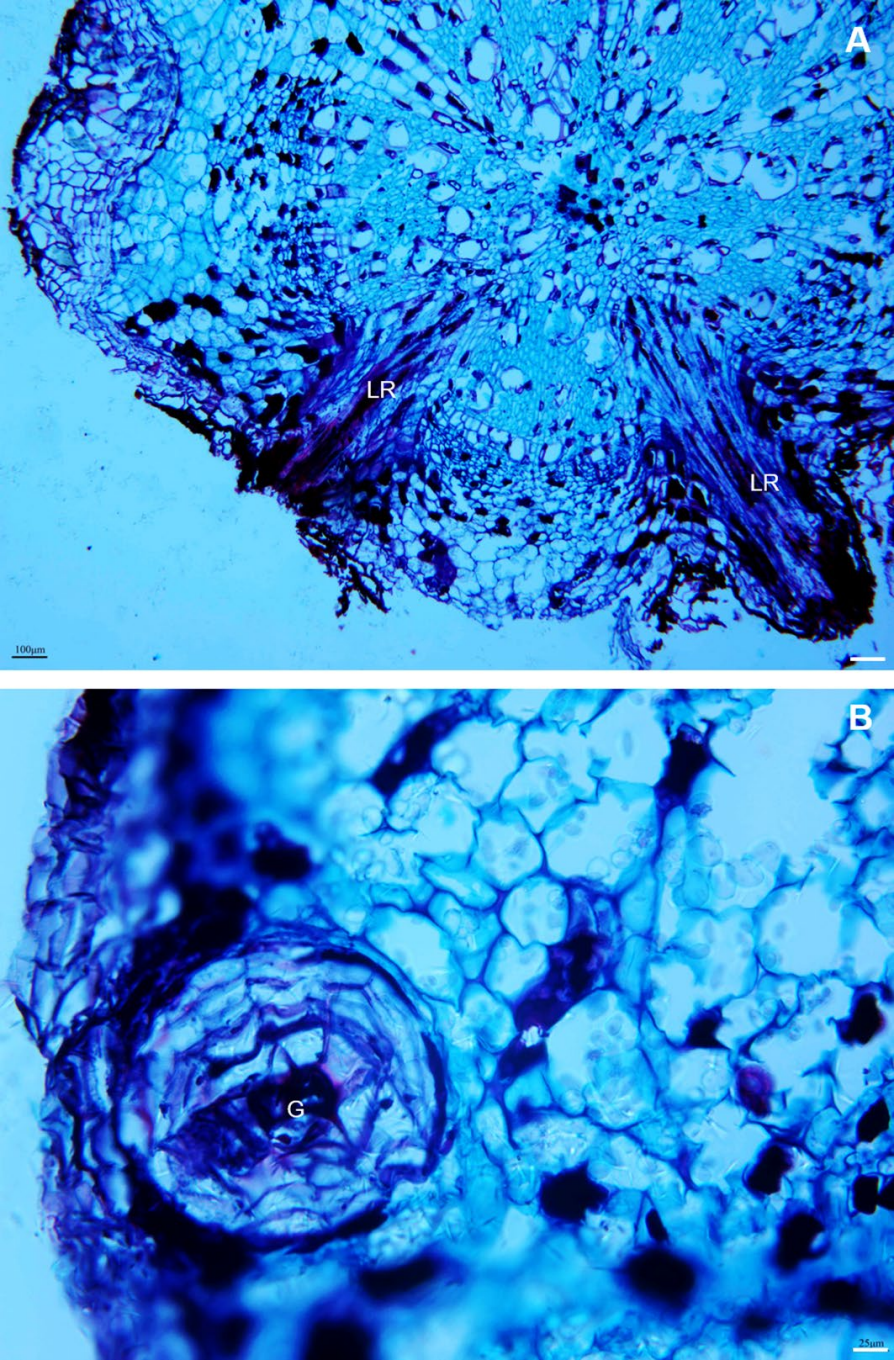
Transverse light micrographs of (**A**) Ca^2+^-deficient roots showing lateral root formation arising from the central vascular stele and (**B**) Ca^2+^-deficient roots showing duct/gland-like structures in the cortex region. Scale bars: 100 µm (**A**), 25 µm (**B**). *G* gland, *LR* lateral roots.

**Figure 8:**
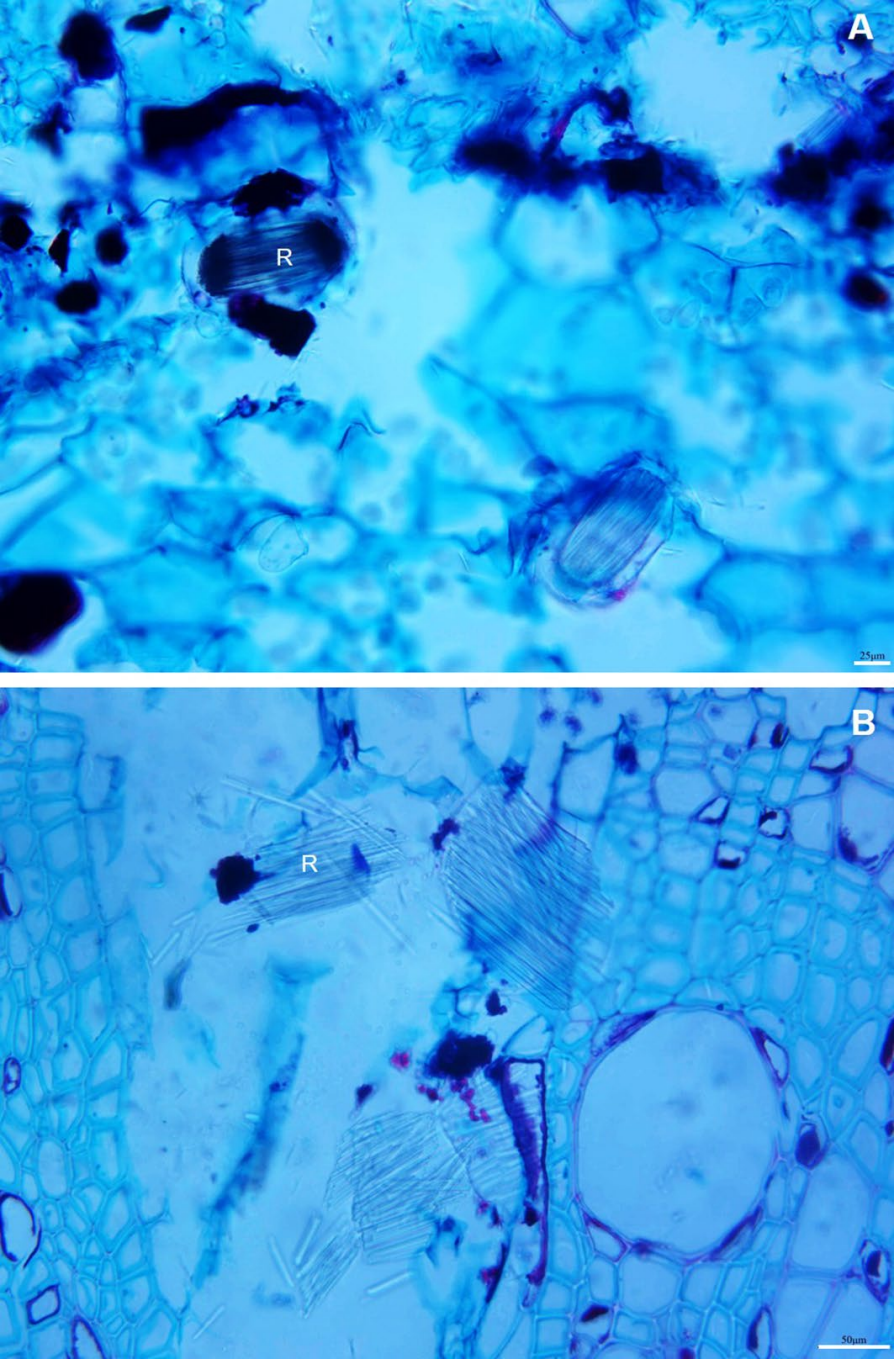
Transverse light micrographs of intact calcium oxalate crystals in (**A**) Ca^2+^-deficient and dissolved crystals in (**B**) Ca^2+^-sufficient roots. Scale bars: 50 µm (**A**), 25 µm (**B**). *R* raphide crystals.

From the anatomical perspective, the root xylem vessels of Ca^2+^-sufficient vines were almost circular and large but fewer than Ca^2+^-deficient roots (Fig. 6A; Table 4). They were small, polygonal, and appeared to be collapsed in the Ca^2+^-deficient roots (Fig. 6B), yet developed with thick cell walls (Table 4). It is noteworthy that despite such morphological changes, the Ca^2+^-deficient roots showed an increased propensity for branching (lateral roots) (Fig. 7A). Also, it developed duct/gland-like structures not observed in the Ca^2+^-sufficient roots (Fig. 7B). Both Ca^2+^-deficient and sufficient roots showed raphide crystals (Fig. 8). The crystals were intact in the Ca^2+^-deficient roots (Fig. 8A), whereas they were dissolved in the Ca^2+^-deficient roots (Fig. 8B). Primarily, Ca^2+^-deficiency induced the roots to be less dense, dark, and necrotic with small polygonal collapsed xylem vessels, yet increased in number and developed lateral roots.

**Table 4:** Root vessel morphology of Ca^2+^-sufficient and Ca^2+^-deficient grapevines.

Treatment	Diameter	Area	Number	Cell wall thickness
Ca sufficiency	60.85 ± 7.72*	2574.65 ± 779.56*	81 ± 3.61	3.49 ± 0.92
Ca deficiency	48.53 ± 0.18	1898.73 ± 376.16	85 ± 4.04*	5.44 ± 0.62*

*Means within a column followed by the same letter are not significantly different according to Students’ *t* test (P < 0.05).

## Discussion

One of the crucial aspects of Ca^2+^ nutrition that remains a mystery is how it influences plants’ growth, structure, and productivity^4^. Consequently, Ca^2+^-deficiency disorders continue to blight the horticultural industry. To better understand the virtues of calcium nutrition, we need to understand how Ca^2+^ transport and function are integrated from the whole plant to the subcellular level in different species^26^. Against this backdrop, we present an in-depth characterization and fundamental understanding of organs’ symptomatology at the whole plant and cellular levels to enhance major fruit crops’ nutritional quality. Such information is necessary to understand the function and the mobility of Ca^2+^ at both the cellular and whole plant level, to further the understanding of signaling, and eventually to design more nutritional crop plants that are also more resilient to stress^26,27^.

The conflicting results from various studies illustrate the complexity of Ca^2+^ nutrition and its functions in plants’ growth and development being enigmatic^3^. Although occasionally, these concerns have been dealt with in different species^5,7^, such erratic and feeble attempts are not of much use in resolving such intermittently pursued issues. To move forward, we need a simple technique applicable to all plants envisaging Ca^2+^ activity in the cytosol and its distribution in different parts of the plant^28^. In this regard, examining symptomatology appears promising in gaining a piece of complete knowledge about Ca^2+^ functionality in different species.

Regardless of species, most Ca^2+^ is accumulated in the faster-growing organs such as the fruit, especially the leaves, after taking into the root system^15^. Consequently, both will be symptomatic in the absence of Ca^2+^, as observed in this study showing chlorosis (Fig. 2B) and other studies with coffee^29^ and *Populus*^9^ plants. Despite being chlorotic, the Ca^2+^-deficient vines exhibited increases in fresh and dry weights of their canes and roots (Table 1). This pattern contradicts other species such as the trifoliate rootstock seedlings (*Poncirus trifoliate* L.), wherein Ca deficiency significantly decreased the fresh and dry weight of root, stem, and leaves^30^. The chlorosis is due to the disintegration of chloroplasts by starch accumulation^10,31,32^, given that Ca^2+^ is needed for exporting photosynthates out of leaves to other growing organs^9^. On the other hand, the reduced leaf area is associated with the altered morphology of mesophyll (palisade parenchyma) cells leading to reduced photosynthesis in the Ca^2+^-deficient vines^10^. It does so by decreasing carboxylation efficiency, photosynthetic capacity, quantum yield^10,33^ and disrupting the PS II system^29^. Furthermore, since leaves are the source of photosynthates^11^ and Ca^2+^ promotes cell elongation and cell division^3^, the reduced capacity of Ca^2+^-deficient leaves resulted in inhibition of shoot length and leaf area. Conversely, stem diameter did not differ (Fig. 1C), similar to tomato stems^34^. It was reduced in cowpea, an annual legume^22^, indicating that the radial growth continued in the grapevine while the cell elongation was inhibited. Furthermore, the cell walls of the root, including the leaf xylem vessels, increased under Ca deficiency (Tables 2, 4), which could be due to different changes in the degree of methyl esterification of pectin and glycoprotein of the cell wall^30^. Another striking feature of grapevines was that they endure Ca^2+^-deficiency much longer than other species such as *Populus* plants^9^. These diverse responses by different species are a testimony to the genetic specificity of utilizing and tolerating low levels of Ca^2+^^16^, and that grapevines could be used as a model plant to reveal all of the functionality of Ca^2+^.

Ca^2+^-deficiency reduced leaf area (Fig. 1B); nonetheless, the anatomical organization was unperturbed (Fig. 3B) even though the mesophyll tissues accumulate most Ca^2+^^15^. As a result, both treatments exhibited morpho-anatomy typical of grapevine leaves^35,36^. Despite the structural similarities, the Ca^2+^-deficient leaves were thicker than Ca^2+^-sufficient leaves yet less active photosynthetically^10^. Also, the Ca^2+^-deficient leaves developed xylem vessels with thick walls (Tables 2, 4), which could be due to different changes in the degree of methyl esterification of pectin and glycoprotein of the cell wall^30^. These consequences contrast with tomato leaves, which showed no difference between Ca^2+^-deficient and Ca^2+^-sufficient leaves^34^. Another interesting feature was that Ca^2+^-deficient leaves were lignified (Fig. 3B) than the Ca^2+^-sufficient leaves, as evident from their intense staining of the vasculature and mesophyll tissues. It has also been observed in other Ca^2+^-deficient species but different organs such as roots with different consequences. For instance, root growth restriction due to Ca^2+^ deficiency activates lignification enzymes such as phenylalanine ammonia-lyase and peroxidases^37^, leading to necrosis of roots as in *Pinus taeda*^38^ and tomato^39^, including the grapevines in this study. Unlike these plants, tomato stems become stiff and woody^39^.

Although stem diameter was not affected, the ultrastructure of tissues in its internodes, such as the chlorenchyma, phloem, and cambium, underwent structural modifications (Fig. 4). This occurrence contrasts with the field peas’ (*Pisum sativum*) stems and roots that showed no variation in their internal structure between Ca^2+^-deficient and Ca^2+^-sufficient plants^40^. More specifically, the Ca^2+^-sufficient cambium cells developed dense cytoplasm (Fig. 4B). Interestingly, quite the contrary occurred in other species such as poplar; their cambium showed the same features under Ca^2+^-deficient conditions^41^. On the other hand, the ultrastructure of Ca^2+^-deficient cambium cells of grapevine was utterly different (Fig. 4A). It exhibited the features of the beginning of the breakdown of the cytoplasm, disintegration of the plasmalemma and tonoplast, and the accumulation of degenerative vesicles^19^. Such processes were initiated due to a lack of unloading of assimilates into the cambium^9^ and were evident from the parietal layer of electron opaque cytoplasm and protuberances. Another striking feature was its strong vacuolation (Fig. 4A), a prerequisite for developing xylem vessels^42^. Accordingly, the Ca^2+^-deficient cambium with one large central vacuole should have relatively developed more xylem vessels. However, the stem diameter (Fig. 1C), which increases due to adding xylem vessels via secondary growth^43^, contradicts this premise as it did not vary between the Ca^2+^-deficient and Ca^2+^-sufficient vines. This means that secondary growth can also be induced by other processes such as high osmotic pressure in cambial cells^12^ and increased cell enlargement and differentiation in the secondary xylem as found in Ca^2+^-deficient *Pinus taeda*^38^*.* On the country, Venning^44^ found a reduction in cambial activity and secondary xylem in calcium-deficient tomato (*Lycopersicon *spp.) plants, reflecting calcium's enzymatic role in the meristematic regions of the plants. As opposed to stem diameter, the shoot length (Fig. 1A), resulting from internodal elongation^45^, was much higher in Ca^2+^-sufficient than in Ca^2+^-deficient vines indicating that Ca^2+^ is required for cell elongation not only in stems but also roots^18^. These studies collectively corroborate that the symptomatology of Ca^2+^-deficiency is not uniform; it varies from species to species. Because of this reason, the Ca^2+^ deficiency symptomatology for different species needs to be defined independently for improving their productivity rather than drawing on from other unrelated species.

Both chloroplasts and mitochondria are indispensable for providing energy and carbon sources to cells and are the major players in various physiological processes, including possessing calcium signals and being the site of critical metabolic pathways^46,47^. In this study, the Ca^2+^-sufficient stem chloroplasts (Fig. 4F) similar to the healthy grapevine^10^, cotton^48^, and citrus^11^ leaves differed from Ca^2+^-deficient stem chloroplasts. For instance, the chloroplast envelope remained intact, unlike the ruptured one in the Ca^2+^-deficient maize leaves^49^. Furthermore, Ca^2+^-deficient stem chloroplasts (Fig. 4E) did not exhibit any plastoglobuli known to occur in healthy stem chloroplasts^50^. How these features compare to stem chloroplasts of other species is not known. Nonetheless, one aspect that is clear in this respect is that Ca^2+^ starvation reduces the unloading of assimilates, for instance, into the stem^9^, similar to found with Ca^2+^-deficient grapevine leaves in which chloroplasts’ membrane assembly (grana and stroma lamellae) disoriented and destroyed due to large starch granules^10^. Regardless, the ultimate result of chloroplast destruction is chlorosis^10^, which explains the Ca^2+^ deficient vines’ chlorotic leaves (Fig. 2B). Furthermore, the malformed chloroplasts under Ca^2+^ deficiency indicated that Ca^2+^ is required for their formation and maintenance^18^ and to store excess intracellular Ca^2+^ in there^1^.

Akin to the leaf chloroplasts, stem chloroplasts also photosynthesize by re-fixing respiratory CO_2_ reducing the CO_2_ concentration with the concomitant increase in oxygen to continue mitochondrial respiration^51^. Such performance depends on the extent of granal density and stacking of grana, starch grains, and plastoglobuli^19,50,52^, which was much higher in the Ca^2+^-sufficient stems (Fig. 4F). Consequently, the Ca^2+^-sufficient stems with their intact phloem tissues (Fig. 4C) are expected to balance photosynthesis and mitochondrial respiration, providing all essential metabolic functions for the whole plant. With no mitochondria formation, which occurs under Ca^2+^ deficiency^6,10^, and the altered phloem tissues (Fig. 4D), the metabolic activity is compromised in Ca^2+^ deficient vines similar to found in Ca^2+^-deficient tomato leaves^53^. One manifestation of such a decline in metabolic activity is the differential accumulation of nutrients required for carrying out numerous physiological processes^51^.

The nutrient concentrations, N, P, K, Ca, Mg, Fe, Mn, Zn, Cu, and B, varied among the roots, trunk, canes, and leaves (Fig. 5). Their differential uptake pattern emphasized that Ca^2+^ in the medium is necessary for root development^6,54^. Also, that uptake of nutrients occurs via selective uptake mechanisms under nutrient sufficiency and deficiency^55^. This aspect is evident from the increased uptakes in Ca^2+^-deficient vines, representing compensation to maintain the cells’ electrical and chemical balance^5^. For instance, the cations, Ca^2+^, Mg^2+^, K^+^ substitute for each other in case of lack or excess of one of them^29^. If accumulated in excess, they interfere with the physiological process^9^. Their reductions arise from competitive interactions or membranes’ ion selectivity^29^. In particular, Cu was reduced in all organs (Fig. 5); Cu uptake is a metabolically mediated process^56^, and the metabolic activity was significantly reduced in Ca^2+^-deficient grapevines^10^.

The Ca^2+^-deficient roots were primarily dark and necrotic (Fig. 2D). The reason is that the absence of Ca^2+^ from an otherwise complete nutrient solution exposes the roots to a highly toxic environment of potassium, magnesium, and other micronutrient ions^6^. Despite the morphological changes, the basic anatomical organization did not change (Fig. 6), also found in *Pisum sativum*^52^. What is noteworthy is that roots developed with more xylem vessels with thick cell walls (Table 4), attributable to an increase in Boron uptake^57^. Also, Ca^2+^ deficiency triggered the formation of lateral roots (Fig. 7A), which typically originate from pericycle cells mediated by auxin^58^. This reaction was perhaps a compensatory response to increase the absorption surface area induced by the increased allocation of photosynthates^59^, which explains the increases in fresh and dry weights of roots in the Ca^2+^-deficient grapevines. Also, resin duct/gland-like structures, including raphide crystals, which typically disappear under Ca^2+^ deficiency^60^, were observed in the Ca^2+^-deficient roots (Figs. 7B, 8A). Unlike in the Ca^2+^-sufficient roots, the raphide crystals were intact in the Ca^2+^-deficient roots indicating that they were older and formed before introducing Ca^2+^ deficiency^60^. This premise is based on the fact that the newly formed crystals typically dissolve^61,62^.

## Conclusions

Ca^2+^ deficiency altered the growth characteristics and organ structures. It reduced shoot length, shoot and trunk fresh weights, leaf area, chlorophyll, and root density causing drooping, yellowing, and chlorosis of leaves. Anatomically, although the roots developed lateral roots, they formed small polygonal xylem vessels. On the other hand, the leaves maintained their anatomical architecture wherein the xylem vessels with thick walls increased, and the mesophyll and vascular tissues were lignified. Ultrastructurally, the chloroplasts, phloem tissues, and cambium displayed significant modifications. These changes were accompanied by significant variations in nutrients among the roots, trunk, canes, and leaves. Although this study provided compelling evidence for Ca^2+^-deficiency symptomatology in divulging some of the fundamental processes of its uptake, utilization, and function, further studies are needed for a holistic understanding of Ca^2+^ functionality and its mode of action in various crops and their genotypes.

## Materials and methods

**Figure 9:**
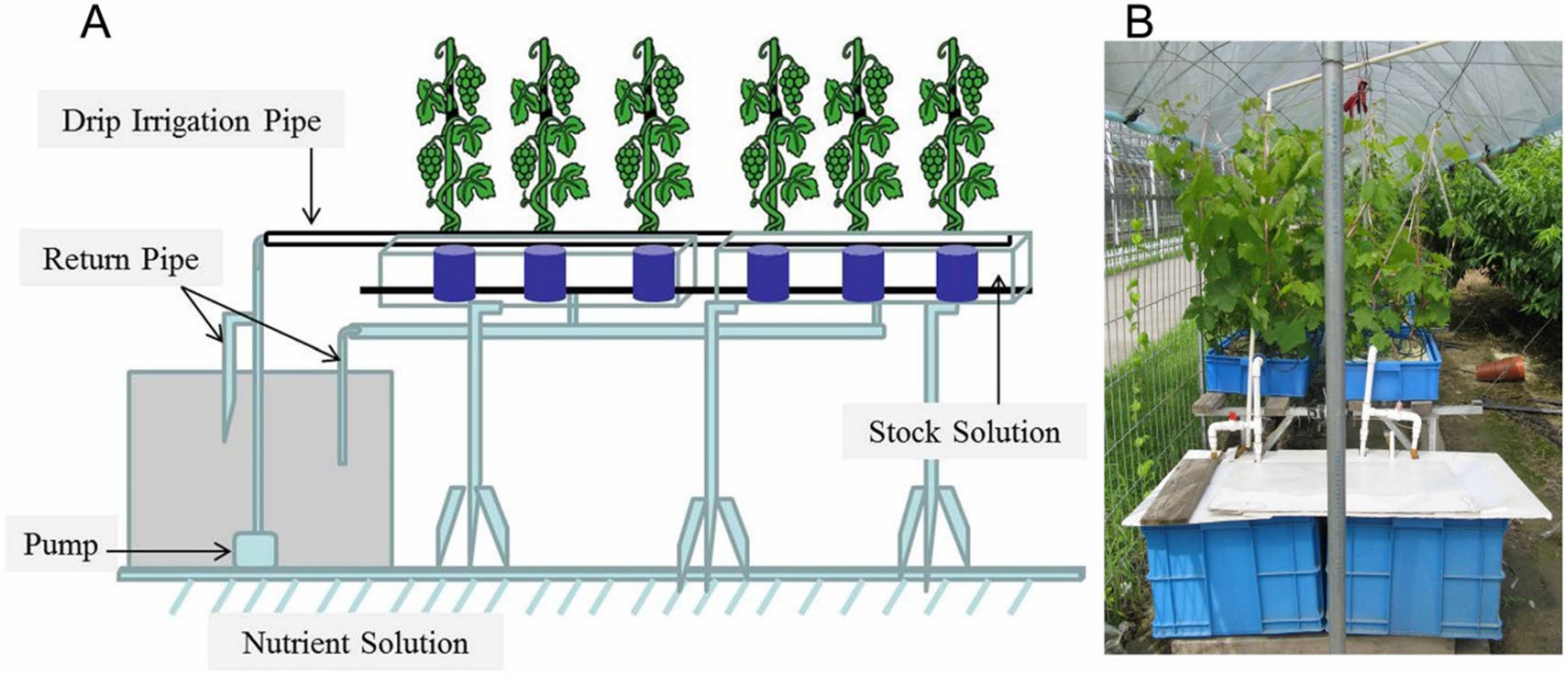
Experimental layout to induce Ca^2+^ deficiency and Ca^2+^ sufficiency in grapevines under greenhouse conditions.

We used potted grapevines fed with nutrient solutions to induce deficiency symptomatology (Fig. 9). Potted plants fed with nutrient solutions are the right approach to this scenario, and it has been applied to several species, for they allow more rigorous control of the composition of the solution, which is to supply the plants’ roots^9^.

One-year-old homogenously rooted Thompson seedless grapevine (*Vitis vinifera* L.) cuttings, uniform in size, were self-grown in hydroponic containers for 6 wk in a glasshouse at 22 °C:18 °C with 16 h:8 h photoperiod. This study complied with relevant institutional and national guidelines. The water utilized for the hydroponic system was ultra-pure grade water (HYZ-20I, Hengnuo water treatment, Chongqing, China), which had no (0 mM) calcium. The substrate in the containers included perlites washed with ultra-pure grade water three to four times before potting the grapevines. All grapevines were irrigated with ultra-pure grade water until the treatments were imposed. The glasshouse was located at Shanghai Jiaotong University, Eastern China (31°13′30.03″N, 121°19′33.59″E). All vines were provided with macro-and micro-nutrients using a modified aerated Hoagland solution (Hoagland and Arnon, 1950) containing 15 mM N (Ca(NO_3_)_2_·4H_2_O 4, KNO_3_ 6, NH_4_H_2_PO_4_ 1, (NH_4_)_6_Mo_7_O_24_·4H_2_O 0.1), 1 mM P (NH_4_H_2_PO_4_), 6 mM K (KNO_3_), 4 mM Ca (Ca(NO_3_)_2_·4H_2_O), 2 mM Mg (MgSO_4_·7H_2_O), 2 mM S (MgSO_4_·7H_2_O), 0.11 mM Fe (Na_2_Fe-EDTA), 0.05 mM B (H_3_BO_3_), 0.01 mM Mn (MnCl_2_·4H_2_O), 0.77 μM Zn (ZnSO_4_·7H_2_O), 0.31 μM Cu (CuSO_4_·5H_2_O), 0.1 μM Mo (NH_4_)6Mo_7_O_24_·4H_2_O) at pH 5.7–6.0 and renewed every 3 days. The respective salts of each nutrient are shown in parenthesis. Ca (NO_3_)_2_ was used as a source of calcium in the Hoagland solution for generating Ca^2+^ sufficient vines, which had a concentration of 4 mM. On the other hand, the Ca^2+^ starved grapevines were applied with NH_4_NO_3_ (4 mM) instead of Ca(NO_3_) in the Hoagland solution (NO_3_) to maintain the ionic balance as well as to induce calcium deficiency as per the studies by Volk et al. (2002)^63^. The experiment was conducted in a randomized block design, lasting for 52 days. Each treatment consisted of 12 vines, and the measurements were made on 6 plants.

The choice of no calcium and sufficient calcium was based on our preliminary experiments, which showed that of all nutrients, a lack of calcium led to the death of the vines. The deficiency of other nutrients induced physiological disorders. That is because calcium regulates almost all our bodily and plant functions by serving both as a nutrient and a messenger^4^. Even though calcium is classified as an essential macronutrient, its requirement is that of a micronutrient (micromoles)^3,4^. Nonetheless, its deficiency causes a severe reduction in the growth and development of plants, including the death of the plants, and the symptomatology varies from species to species, and plants have no adaptive mechanisms to calcium deficiency, unlike other nutrients such as potassium^64^. Consequently, calcium-deficient plants suffer the most^21^.

### Growth characteristics and chlorophyll

The shoot length and diameter were measured weekly 10 days after imposing the treatments, i.e., Ca^2+^ deficiency and Ca^2+^ sufficiency. Leaf area was measured 52 DAT (days after treatment) using an LI-3000A leaf area scanner (LiCor Inc. Lincoln, NB, USA). More specifically, only the midvein length was measured at weekly intervals to measure the leaf area during the experiment. After that, at 52 DAT, the leaves were sampled to scan the leaf areas, and a linear regression equation was used to compute the leaf areas for the whole experiment. Since grapevine leaves attain maximum physiological activity at 40 days of age and start to decline at about 52 days^65^, we lasted the experiment to 52 days. Chlorophyll was measured using a SPAD-502 chlorophyll meter (Zhejiang Top Cloud-Agri Technology Co., Ltd., Hangzhou, China).

### Nutrient analysis

Six vines were separated into root, trunk, cane, stem, and leaf at 52 DAT for each treatment. The separated parts were oven-dried to constant weight in an oven at 80 C and ground for nutrient analysis. According to the Kjeldahl digestion method^66^, the N content was determined by Kjeltec 2300 Analyzer (Kjeltec Analyzer Unit, Foss Tecator, Sweden). The P, K, Ca, Mg, Fe, Zn, B, Cu, Mn content were measured by ICP-AES (ICP-5000, Thermo Fisher Scientific, Inc., NY, USA).

### Leaf and root anatomy

Several small pieces of roots and leaf sections (1 × 1 cm) were cut from mid-laminar areas of Ca^2+^ deficient and sufficient vines using a razor blade and fixed in FAA (90 ml 70% alcohol: 5 ml glacial acetic acid: 5 ml 40% formaldehyde). Samples were vacuumed 1 h and stored at 4 °C until the samples were further processed using the microwave paraffin technique^67^. Before embedding, all steps entailing fixation were performed at 75 °C in a water bath. The fixative in the vial was replaced twice with a new pre-chilled solution for 2 min each time. The fixed samples were dehydrated with 60% ethanol for 2 min for dehydration, followed by 50% ethanol and 50% Tert-butanol for 2 min. For infiltration, the samples were first treated with 100% Tert-butanol for 7–8 min and then 50% Tert-butanol/50% paraffin for 2 min. Subsequently, the specimens were microwaved in 100% paraffin wax for 7.5 min and embedded and cooled to room temperature. After cooling, the paraffin blocks were sliced at eight μm and placed on a slide warmer at 48 °C for 24–48 h. The sections were stained with double staining of Safranine T and fast green. When staining was complete, a drop of mounting medium (Canadian neutral gum) was used to affix coverslips to the slides. Slides were placed under a compound microscope (Olympus BX43, Olympus Co., Tokyo, Japan) attached with a digital camera to capture digital images, and the anatomical measurements were performed using the cellSens imaging software platform.

### Leaf ultrastructure

The method described by Xie et al. (2009)^61^ was adopted to examine the leaf ultrastructure. To examine the leaf ultrastructure, several 0.5 cm × 0.5 cm pieces were cut from mid-laminar areas of both leaves, using a razor blade, fixed in 0.25% glutaraldehyde in 0.2 M sodium phosphate buffer at pH 7.0. Samples were vacuumed for 1 h and stored overnight at 4 °C. The fixed tissues were washed with 0.1 M phosphate buffer and post-fixed in 2% OsO4 for 4–6 h at 4 °C. The leaf samples were dehydrated in a series of ethanol and acetone and embedded in the epoxy resin Epon-812. Ultra-thin sections (50—70 nm) transverse sections were cut with a glass knife and mounted on 200-mesh copper grids. The leaf sections were then double-stained with 2% uranyl acetate and 2.6% lead citrate and examined under a transmission electron microscope (Tecnai G2 Spirit Biotwin, FEI, Hillsboro, USA) (TEM) at 120 kV.

### Statistical analysis

All data were subjected to analysis of variance (ANOVA). Significant differences (P < 0.05) between means were determined using a *t* test. The data were analyzed using SPSS (Version 11, SPSS, Chicago, IL, USA) statistical package.
